# Coupled changes in hippocampal structure and cognitive ability in later life

**DOI:** 10.1002/brb3.838

**Published:** 2018-01-04

**Authors:** Devasuda Anblagan, Maria C. Valdés Hernández, Stuart J. Ritchie, Benjamin S. Aribisala, Natalie A. Royle, Iona F. Hamilton, Simon R. Cox, Alan J. Gow, Alison Pattie, Janie Corley, John M. Starr, Susana Muñoz Maniega, Mark E. Bastin, Ian J. Deary, Joanna M. Wardlaw

**Affiliations:** ^1^ Centre for Cognitive Ageing and Cognitive Epidemiology University of Edinburgh Edinburgh UK; ^2^ Department of Neuroimaging Sciences Centre for Clinical Brain Sciences University of Edinburgh Edinburgh UK; ^3^ Scottish Imaging Network A Platform for Scientific Excellence (SINAPSE) Collaboration Edinburgh UK; ^4^ Edinburgh Dementia Research Centre UK Dementia Research Institute Edinburgh UK; ^5^ Department of Psychology University of Edinburgh Edinburgh UK; ^6^ Department of Computer Science Lagos State University Lagos Nigeria; ^7^ Department of Psychology School of Social Sciences Heriot‐Watt University Edinburgh UK; ^8^ Alzheimer Scotland Dementia Research Centre University of Edinburgh Edinburgh UK

**Keywords:** aging, cognition, hippocampus, magnetic resonance imaging, MRI biomarkers

## Abstract

**Introduction:**

The hippocampus plays an important role in cognitive abilities which often decline with advancing age.

**Methods:**

In a longitudinal study of community‐dwelling adults, we investigated whether there were coupled changes in hippocampal structure and verbal memory, working memory, and processing speed between the ages of 73 (*N* = 655) and 76 years (*N* = 469). Hippocampal structure was indexed by hippocampal volume, hippocampal volume as a percentage of intracranial volume (H_ICV), fractional anisotropy (FA), mean diffusivity (MD), and longitudinal relaxation time (T1).

**Results:**

Mean levels of hippocampal volume, H_ICV, FA, T1, and all three cognitive abilities domains decreased, whereas MD increased, from age 73 to 76. At baseline, higher hippocampal volume was associated with better working memory and verbal memory, but none of these correlations survived correction for multiple comparisons. Higher FA, lower MD, and lower T1 at baseline were associated with better cognitive abilities in all three domains; only the correlation between baseline hippocampal MD and T1, and change in the three cognitive domains, survived correction for multiple comparisons. Individuals with higher hippocampal MD at age 73 experienced a greater decline in all three cognitive abilities between ages 73 and 76. However, no significant associations with changes in cognitive abilities were found with hippocampal volume, FA, and T1 measures at baseline. Similarly, no significant associations were found between cognitive abilities at age 73 and changes in the hippocampal MRI biomarkers between ages 73 and 76.

**Conclusion:**

Our results provide evidence to better understand how the hippocampus ages in healthy adults in relation to the cognitive domains in which it is involved, suggesting that better hippocampal MD at age 73 predicts less relative decline in three important cognitive domains across the next 3 years. It can potentially assist in diagnosing early stages of aging‐related neuropathologies, because in some cases, accelerated decline could predict pathologies.

## 1 INTRODUCTION

The hippocampus plays an important role in cognitive functions such as memory, learning, and spatial navigation (Förster et al., 2012; Muzzio, Kentros, & Kandel, 2009; Nossin‐Manor et al., 2012). Hippocampal volume tends to decrease gradually with age (Scahill et al., 2003), and greater hippocampal volume reduction correlates with steeper pathological cognitive decline and Alzheimer's dementia (A Convit et al., 1997; De Leon et al., 1997; Jack et al., 1998, 1997; Korf, Wahlund, Visser, & Scheltens, 2004; L. A. van de Pol, Hensel, Barkhof et al., 2006). However, the association between the volume of the hippocampus and cognitive performance in nonpathological aging varies between studies (Ferguson, Wardlaw, & MacLullich, 2010), with some showing a significant association (Aribisala et al., 2014; Erickson et al., 2010; van der Lijn, den Heijer, Breteler, & Niessen, 2008; Ystad et al., 2009), and others showing no association (Sánchez‐Benavides et al., 2010). In addition, some studies have shown substantial overlap between hippocampal volume in healthy controls and patients with Alzheimer's disease once adjusted for intracranial capacity, and the range of hippocampal volumes is large in healthy adults (Barnes et al., 2004; Antonio Convit et al., 1993; Lupien et al., 2007; L. van de Pol, Hensel, van der Flier et al., 2006). This suggests that volume alone does not fully indicate hippocampal integrity, and smaller adjusted volumes may not necessarily signify deterioration.

The effect of cellular changes underpinning age‐related brain tissue loss, such as neurodegeneration and synapse loss (Hyman, Van Hoesen, Damasio, & Barnes, 1984), can be investigated using quantitative magnetic resonance imaging (MRI) techniques such as diffusion tensor MRI (DT‐MRI) and relaxometry (Ceccarelli et al., 2007; Cercignani, Bozzali, Iannucci, Comi, & Filippi, 2001; Parry et al., 2003; Vrenken, Rombouts, Pouwels, & Barkhof, 2006). DT‐MRI can be used to measure white and gray matter microstructural changes (Bhagat & Beaulieu, 2004; den Heijer et al., 2012), broadly speaking via two scalar indices, fractional anisotropy (FA) and mean diffusivity (MD). FA signifies the directional dependence of water molecules within cellular boundaries within a tissue, and MD represents the overall magnitude of water diffusion (Le Bihan, 2003). FA is reduced and MD is increased in many pathologies associated with changes in water content, disruption and break down of tissue cytoarchitecture, demyelination, and diseased tissue (Beaulieu, 2002; Bhagat & Beaulieu, 2004; den Heijer et al., 2012; Hsu et al., 2010; Neil, Miller, Mukherjee, & Hüppi, 2002; Pal et al., 2011). Studies have also reported a decrease in FA and increase in MD in older people in parahippocampal white matter and in the hippocampus (Rose et al., 2006; Salat et al., 2010). The longitudinal relaxation time (T1) is, in part, related to brain tissue water content. Increased T1 values indicate increased tissue water content; for example, as seen in peritumoral tissues where there is extracellular edema (Bastin, Sinha, Whittle, & Wardlaw, 2002). Across all ages, T1 is longer in the gray matter and shorter in the white matter of the brain (Saito, Sakai, Ozonoff, & Jara, 2009). A previous study showed that T1 declines throughout adolescence and early adulthood, achieving a minimum value in the fourth to sixth decade of life, and then T1 begins increasing (Cho, Jones, Reddick, Ogg, & Steen, 1997).

In previous analyses including a cross‐sectional sample from the Lothian Birth Cohort 1936 (LBC1936) at age 73 that included 565 participants, higher MD and T1 in the hippocampus were associated with lower fluid intelligence, slower processing speed, and poorer memory, whereas higher FA was associated with higher fluid intelligence and processing speed but not memory (Aribisala et al., 2014; den Heijer et al., 2012). In this study, we expand these findings by investigating the longitudinal relationships between neuroimaging biomarkers and three broad domains of cognitive ability in the LBC1936 between approximately 73 and 76 years of age. The cognitive domains—verbal memory, working memory, and information processing speed—were selected based on the hippocampus's role in memory and processing information; the detailed cognitive testing available in the cohort allowed us to test the potential links between changes in hippocampal morphology and changes in multiple cognitive domains. On the basis of prior work on the hippocampus indicating its stronger role in episodic rather than other types of memory (Moscovitch, Cabeza, Winocur, & Nadel, 2016), we predicted that there would be stronger relations between the hippocampal measures and tests of verbal memory compared with tests of working memory. We used latent variable modeling to minimize cognitive test‐specific measurement error. We assessed the relationship between cognitive changes and changes in general hippocampal volume, hippocampal volume as a percentage of intracranial volume (H_ICV), FA, MD, and T1 over a 3‐year period.

## 2 METHOD

### 2.1 Participants

The LBC1936 is a longitudinal study of community‐dwelling adults in the Edinburgh and Lothians area of Scotland, all of whom were born in 1936. Most of the participants took part in the Scottish Mental Survey 1947 when they were approximately 11 years of age; they have repeatedly returned for cognitive testing and neuroimaging in later life. Participants underwent a series of tests in three sequential waves at mean ages of 69.53 years (*SD* = 0.83 years) in 2004–2007 (*n* = 1, 091, 543 females), 72.49 years (*SD* = 0.71 years) in 2007–2010 (*n* = 866, 418 females), and 76.25 years (*SD* = 0.68 years) in 2011–2014 (*n* = 697, 337 females). Written informed consent was obtained from all participants before testing. Full details of the cohort are available elsewhere (Deary, Gow, Pattie, & Starr, 2012; Deary et al., 2007). The LBC1936 study was approved by the Multi‐Centre Research Ethics Committee for Scotland (MREC/01/0/56), the Lothian Research Ethics Committee (LREC/2003/2/29), and the Scotland A Research Ethics Committee (second and third waves: 07/MRE00/58).

This study uses data from the second and third waves, in which both cognitive testing and brain MRI were conducted; neuroimaging brain data were not collected at the first wave. Cognitive testing was conducted at a different visit to brain MRI, with an average of 65.04 days (*SD* = 39.57 days) between sessions at the second wave, and 40.29 days (*SD* = 31.89 days) at the third wave. A total of 731 participants underwent brain MRI at the second wave (mean age 72.68, *SD* 0.72 years), and 488 at the third wave (mean age 76.38, *SD* 0.65 years) of the study. Not all participants provided sufficient or usable data; valid sample sizes for each brain measure are shown in Table 1. Hippocampal imaging data were available from 655 participants (309 females) at the second wave, and 469 participants (218 females) at the third wave. We used all available data in the analyses.

**Table 1:** Descriptive statistics of the sample, including hippocampal MRI biomarker measurements and cognitive variables used in the analysis

Variable type	Variables	Wave 2 (age ~73 years)			Wave 3 (age ~76 years)		
		*n*	Mean	*SD*	*n*	Mean	*SD*
Demographic	Age (years)	655 (346M, 309F)	72.50	0.71	469 (251M, 218F)	76.24	0.65
Hippocampal measures	Volume (mm^3^)	655	6429.53	861.22	469	5634.92	914.52
	Percentage volume (%)	643	0.48	0.05	464	0.39	0.06
	FA	636	0.12	0.01	458	0.11	0.01
	MD (×10^−3^ mm^2^s^−1^)	636	0.88	0.05	458	0.93	0.05
	T1 (s)	653	1.66	0.15	442	1.44	0.19
Cognitive tests	Logical memory	864	74.23	17.89	688	74.58	19.20
	Verbal paired associates	843	27.18	9.49	663	26.41	9.56
	Spatial Span	861	14.69	2.76	690	14.62	2.73
	Digit Span Backward	866	7.81	2.29	695	7.77	2.37
	Letter‐Number Sequencing	863	10.91	3.08	687	10.48	2.99
	Digit‐Symbol Substitution	862	56.40	12.31	687	53.81	12.93
	Symbol Search	862	24.61	6.18	685	24.60	6.46
	Choice Reaction Time	865	0.65	0.09	685	0.68	0.10
	Inspection Time	838	111.22	11.79	654	110.17	12.53

Values for hippocampal measures come from the average across both hippocampi. MD values were multiplied by 10^3^ before inclusion in the table.

### 2.2 Brain MRI Acquisition

Full details of the neuroimaging protocol are described elsewhere (Wardlaw et al., 2011). Briefly, the second and third waves of the study employed an identical imaging protocol using the same 1.5 T GE Signa Horizon HDxt clinical scanner (General Electric, Milwaukee, WI, USA) with a self‐shielding gradient set with maximum gradient strength of 33 mT/m and an eight‐channel phased‐array head coil. The MRI scanner is maintained on a careful quality assurance program. The structural imaging included a high‐resolution 3D T1‐weighted volume, T2‐weighted, T2*‐weighted, and fluid‐attenuated inversion recovery (FLAIR) scans of the whole brain.

The whole‐brain DT‐MRI acquisition consisted of seven T2‐weighted (*b*_*0*_ = 0 s/mm^2^) and sets of diffusion‐weighted (*b *=* *1,000 s/mm^2^) single‐shot spin‐echo planar imaging (EPI) volumes acquired with 64 noncollinear diffusion encoding directions (Jones et al., 2002).

Quantitative T1 maps were obtained from two‐axial T1‐weighted fast‐spoiled gradient echo (FSPGR) sequences with 2° and 12° flip angles.

All sequences, except the T1‐weighted volume scan, were acquired in the axial plane with a field of view of 256 × 256 mm^2^. Some imaging parameters varied for the different acquisitions: imaging matrix (128 × 128 for DT‐MRI and 256 × 256 for all other acquisitions), and contiguous slice locations and slice thickness (160 × 1.3 mm for high‐resolution T1‐weighted volumes, 36 × 4 mm for FLAIR, and 72 × 2 mm for all other acquisitions, respectively). These parameters were selected to ease co‐registration between sequences, so that FA, MD, and T1 biomarkers could be accurately measured in the hippocampus between individuals and across time.

### 2.3 Image analysis

All image analysis was performed blind to clinical and nonclinical characteristics (including cognitive ability measures) of participants at the second and third waves. Using tools from the FMRIB Software Library version 4.1 (http://www.ndcn.ox.ac.uk/divisions/fmrib/) (SUSAN (Smith & Brady, 1997), FLIRT (Jenkinson, Bannister, Brady, & Smith, 2002) and FIRST (Patenaude, Smith, Kennedy, & Jenkinson, 2011)) and an age‐relevant template (Farrell et al., 2009), initial segmentations of hippocampal structures were generated from high‐resolution T1‐weighted volumes following a previously established pipeline (Wardlaw et al., 2011). These segmentations were visually inspected and, where necessary, manually edited and saved as binary masks by an experienced image analyst using Analyze 10.0 (Mayo Clinic, Rochester, MN, USA; www.analyzedirect.com). These masks were used to compute hippocampal volume measurements for each participant. This procedure complies with a previously established standard hippocampal segmentation protocol (Boccardi et al., 2015). Intracranial volume (ICV; consisting of soft tissue structures inside the cranial cavity including brain, cerebrospinal fluid, dura, and venous sinuses), gray matter, and normal appearing white matter were semi‐automatically segmented using a multispectral image‐processing tool (Valdés Hernández, Ferguson, Chappell, & Wardlaw, 2010) and, where necessary, manually edited using Analyze 10.0. Hippocampal volume as a percentage of intracranial volume (H_ICV) was computed.

DT‐MRI data were preprocessed using FSL tools (FMRIB, Oxford, UK; http://www.ndcn.ox.ac.uk/divisions/fmrib/fsl). This included brain extraction and removal of bulk participant motion and eddy current‐induced artifacts by registering the diffusion‐weighted to the first undistorted T2‐weighted EPI volume for each subject. FA and MD parametric maps were generated using DTIFIT. For each dataset, nonlinear registration facilitated by the TractoR software package (www.tractor-mri.org.uk/diffusion-processing) (Clayden et al., 2011; Modat et al., 2010) was used to obtain the transformation between the brain‐extracted structural T2‐weighted volume and the T2‐weighted (*b*_*o*_) EPI volume, for both baseline and follow‐up. These transformation matrices were then applied to the hippocampal masks. Subsequently, the hippocampal masks were then applied to FA and MD maps, and the median values of FA and MD within the hippocampal structure were computed for each time point.

Quantitative T1 maps were generated on a voxel by voxel basis from the 2° and 12° flip angle T1‐weighted FSPGR volumes as previously described (Armitage, Schwindack, Bastin, & Whittle, 2007; Wardlaw et al., 2011). FLIRT was used to transform the high‐resolution T1‐weighted volume scan into the native space of the quantitative T1 parametric maps. These transformation matrices were then applied to the hippocampal masks to obtain median values of T1 within the hippocampal structures.

**Figure 1:**
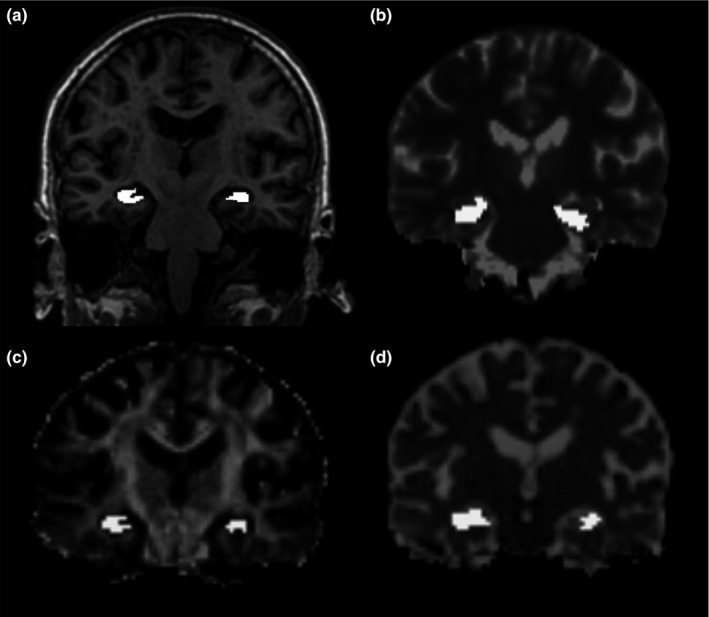
The left and right hippocampal mask overlaid on (a) T1‐weighted volume and maps of (b) T1, (c) FA, and (d) MD in the same participant

An experienced image analyst (DA) visually assessed the overlays of hippocampal masks in the FA, MD, and T1 parametric maps before finalizing the median values of the hippocampal structure for each subject; see Figure 1.

Before modeling, all hippocampal variables were controlled for sex and age in days at scanning. This was achieved by saving the residuals from a linear regression model with each hippocampal variable as the outcome, and sex and age as predictors.

### 2.4 Cognitive ability assessments

All participants completed 16 cognitive ability measures at each wave; a selection of these were used in this study since they related to different memory functions (a key role of the hippocampus) and information processing speed (a cognitive domain theoretically and empirically linked to DT‐MRI measures (Penke et al., 2010)). All cognitive tests were administered in an identical manner in both waves of the study. Three latent factors were calculated at each age to indicate three important cognitive domains. First, *Verbal Memory* was indicated by total scores from the immediate and delayed Logical Memory and Verbal Paired Associates subtests of the Wechsler Memory Scale, Third UK Edition (WMS‐III^UK^) (Wechsler, 1998). Second, *Working Memory* was indicated by total scores from the WMS‐III^UK^ Spatial Span (forwards and backwards), and the Digit Span Backward, and Letter‐Number Sequencing subtests of the Wechsler Adult Intelligence Scale, 3rd UK Edition (WAIS‐III^UK^) (Wechsler, 1998). Finally, *Processing Speed* consisted of the following four assessments, which were a combination of clerical, experimental psychology‐derived, and psychophysics‐derived tasks, assessing speed from a variety of perspectives: WAIS‐III^UK^ Digit‐Symbol Substitution, Symbol Search (both speeded pencil‐and‐paper tasks), and tests of 4‐Choice Reaction Time (measured on a dedicated instrument (Deary, Der, & Ford, 2001)), and Inspection Time (a psychophysical test of perceptual discrimination (Deary et al., 2004)). All participants also completed the Mini‐Mental State Examination (MMSE; (Folstein, Folstein, & McHugh, 1975)). This test is scored out of 30 and scores less than 24 are often used to indicate possible cognitive impairment (Filippi & Rovaris, 2000). As with the hippocampal variables, before entry into the models described below, all cognitive variables were residualized for sex and age in days at the time of testing.

### 2.5 Statistical analysis

To estimate the relationship between hippocampal volume and microstructure, and cognitive aging, we implemented longitudinal latent change score structural equation models (McArdle, 2009). These models, estimated using two waves of data, involve the extraction of a change score variable to assess the difference from the initial wave to the follow‐up. They thus allow the calculation of three types of correlation: level‐level correlations (testing the extent to which the variables are related at the initial measurement), level‐change correlations (testing the extent to which the initial level of one variable predicts subsequent change in another), and change‐change correlations (testing the extent to which there is coupled change between the variables).

Here, we estimated five different latent change score models, one for each hippocampal measurement (volume, H_ICV, FA, MD, and T1). For the hippocampal FA, MD, and T1 variables, we averaged across the right and left hemisphere measurements. Note that only the cognitive abilities were estimated using latent variables; the hippocampal measures were manifest variables at both waves and thus did not produce error‐free latent change variables.

The models used full‐information maximum likelihood (FIML) estimation to deal with the missing data. This method allows all of the data to be used to estimate parameters (paths within the models) with minimum bias under the assumption that data are “missing at random” (MAR (Rubin, 1976)). The MAR assumption requires that any systematic attrition from the study is unrelated to the unobserved data. All models were implemented in MPlus version 7.3 (https://www.statmodel.com/) (Muthén & Muthén, 1998).

All cognitive variables were coded such that higher values indicate better performance. Thus, for example, in what follows, negative level‐level correlations indicate that higher levels of the hippocampal variable are related to lower levels of cognitive ability (and vice versa); negative level‐change correlations indicate that higher levels of the baseline variable are related to steeper subsequent decline in the other variable (and vice versa); and negative change‐change correlations indicate that individuals who decline in one variable tend to develop higher levels of the other variable with time (and vice versa).

Finally, given the large number of correlations tested across the five models, some associations may represent false positives (Type I errors). For that reason, we corrected the correlations from the structural section of each model (separately) for multiple comparisons using the False Discovery Rate (FDR) correction (Benjamini & Hochberg, 1995).

## 3 RESULTS

**Figure 2:**
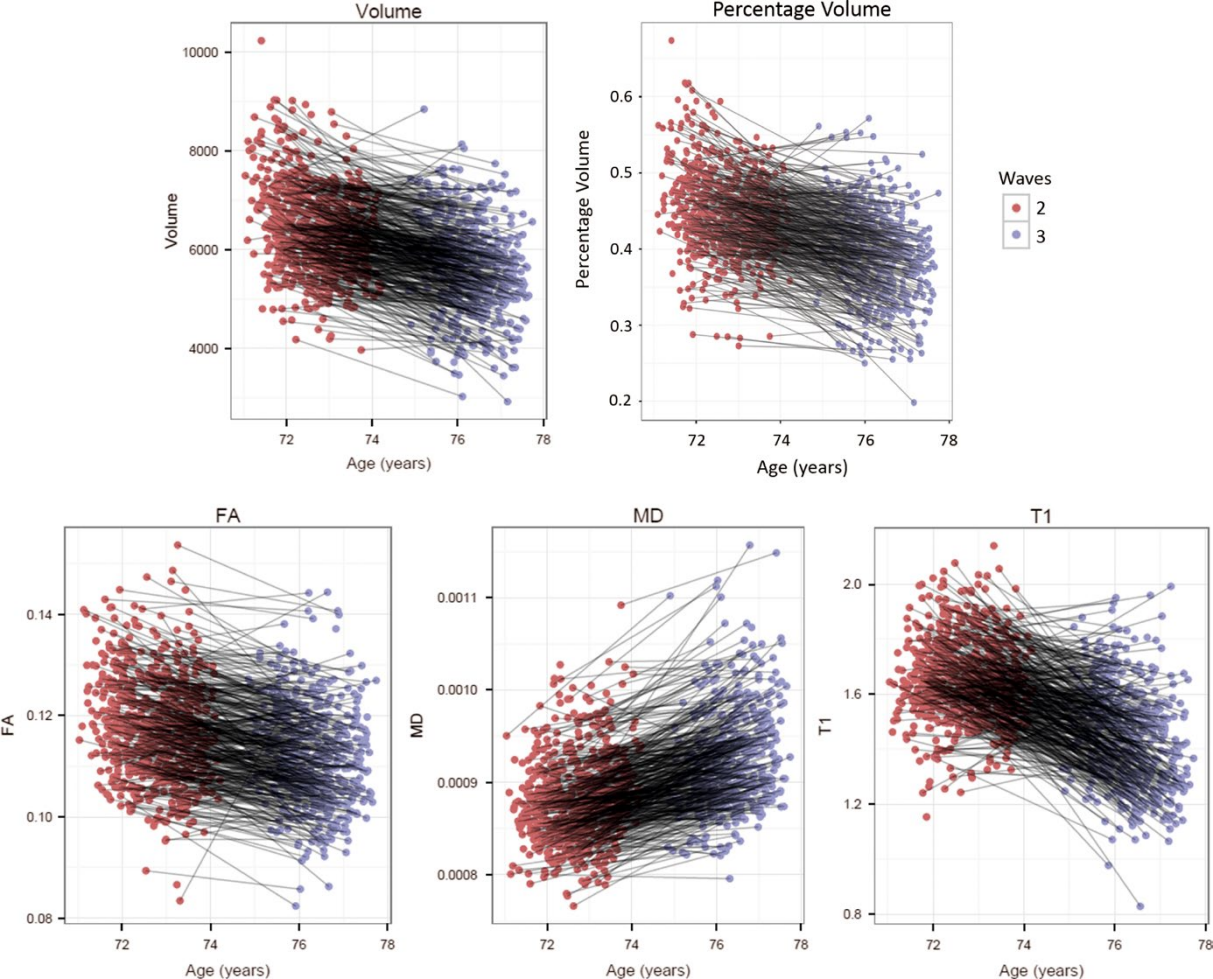
Longitudinal change in each hippocampal variable. Each participant has a single point at the initial scanning wave (mean age: 73; red) and at the follow‐up wave (mean age: 76; purple). Participants who contributed data at both waves have their points connected by a gray line. Volume = hippocampal volume (mm^3^); Percentage Volume = hippocampal volume as a percentage of ICV; FA = hippocampal fractional anisotropy; MD = hippocampal mean diffusivity (mm^2^s^−1^); T1 = hippocampal T1 (s)

Descriptive statistics and a correlation matrix for all variables can be found in Table 1, Figure 2, and Table 2. For the five hippocampal variables, the cross‐wave correlations ranged from Pearson's *r *=* *.19 for T1 to *r *=* *.67 for FA (all *p*‐values <.001). For the individual cognitive tests in the verbal memory, working memory, and processing speed domains, the mean cross‐wave correlations were *r *=* *.99, 0.95, and 0.94, respectively. Those with higher baseline scores at age 73 showed significantly greater decline for processing speed (*r = *−.18, *p *=* *.01) and working memory (*r = *−.21, *p *=* *.01), but not verbal memory (*r *= −.047, *p *=* *.524).

**Table 2:** Pearson correlation matrix for each volumetric measurement, quantitative MRI parameter, and cognitive variable used in the analysis

Variable	1	2	3	4	5	6	7	8	9	10	11	12	13	14	15	16	17	18	19	20	21	22	23	24	25	26	27
1. H Vol 73	‐																										
2. H Vol_ICV 73	0.74	‐																									
3. H FA 73	0.30	0.16	‐																								
4. H MD 73	−0.10	−0.11	−0.33	‐																							
5. H T1 73	−0.05	−0.14	−0.21	0.10	‐																						
6. H Vol 76	**0.64**	0.40	0.24	−0.16	−0.07	‐																					
7. H Vol_ICV 76	0.35	**0.56**	0.08	−0.17	−0.14	0.82	‐																				
8. H FA 76	0.21	−0.03	**0.67**	−0.29	−0.13	0.31	0.14	‐																			
9. H MD 76	−0.23	−0.22	−0.33	**0.66**	0.19	−0.20	−0.18	−0.35	‐																		
10. H T1 76	0.05	0.03	−0.09	0.15	**0.19**	0.06	−0.02	−0.02	−0.03	‐																	
11. LM 73	0.06	0.07	0.09	−0.17	−0.11	0.12	0.07	0.07	−0.20	−0.05	‐																
12. VPA 73	0.02	0.08	0.09	−0.16	−0.14	0.08	0.09	0.09	−0.12	−0.09	0.51	‐															
13. SpS 73	0.12	0.00	0.14	−0.16	−0.06	0.17	0.13	0.13	−0.14	−0.01	0.22	0.18	‐														
14. DSB 73	0.09	0.09	0.12	−0.13	−0.05	0.05	0.11	0.11	−0.06	−0.01	0.29	0.27	0.31	‐													
15. LNS 73	0.08	0.06	0.13	−0.07	−0.14	0.11	0.12	0.12	−0.09	−0.08	0.36	0.30	0.40	0.55	‐												
16. DSS 73	0.04	0.06	0.20	−0.22	−0.14	0.14	0.19	0.19	−0.21	−0.07	0.32	0.26	0.32	0.33	0.43	‐											
17. SSe 73	0.05	−0.01	0.17	−0.19	−0.09	0.14	0.17	0.17	−0.22	−0.06	0.28	0.21	0.39	0.33	0.40	0.63	‐										
18. CRT 73	−0.08	−0.04	−0.16	0.19	0.15	−0.07	0.15	−0.15	0.19	0.09	−0.26	−0.22	−0.35	−0.22	−0.38	−0.55	−0.50	‐									
19. IT 73	0.05	−0.03	0.17	−0.16	−0.11	0.14	0.12	0.12	−0.20	0.04	0.18	0.19	0.29	0.19	0.30	0.38	0.36	−0.38	‐								
20. LM 76	0.10	0.13	0.12	−0.22	−0.11	0.16	0.09	0.09	−0.27	−0.14	**0.73**	0.39	0.18	0.27	0.35	0.33	0.26	−0.22	0.17	‐							
21. VPA 76	0.08	0.15	0.11	−0.15	−0.17	0.10	0.10	0.10	−0.15	−0.12	0.44	**0.70**	0.10	0.28	0.30	0.28	0.19	−0.21	0.17	0.53	‐						
22. SpS 76	0.04	−0.04	0.10	−0.15	−0.09	0.12	0.12	0.12	−0.12	−0.14	0.21	0.15	**0.57**	0.30	0.33	0.29	0.36	−0.31	0.24	0.24	0.17	‐					
23. DSB 76	0.08	0.09	0.09	−0.11	−0.07	0.07	0.12	0.12	−0.07	−0.06	0.26	0.26	0.32	**0.68**	0.52	0.34	0.29	−0.21	0.18	0.31	0.31	0.31	‐				
24. LNS 76	0.06	0.03	0.15	−0.17	−0.06	0.12	0.15	0.15	−0.16	−0.11	0.34	0.25	0.29	0.47	**0.66**	0.41	0.33	−0.31	0.22	0.38	0.34	0.34	0.56	‐			
25. DSS 76	0.04	0.05	0.17	−0.23	−0.12	0.15	0.14	0.14	−0.22	−0.09	0.31	0.21	0.27	0.31	0.40	**0.83**	0.58	−0.53	0.32	0.39	0.28	0.32	0.34	0.42	‐		
26. SSe 76	0.14	0.07	0.19	−0.24	−0.12	0.18	0.21	0.21	−0.29	−0.10	0.30	0.27	0.42	0.35	0.39	0.62	**0.67**	−0.50	0.34	0.34	0.26	0.40	0.35	0.40	0.65	‐	
27. CRT 76	−0.08	−0.10	−0.16	0.25	0.15	−0.13	−0.13	−0.13	0.23	0.13	−0.18	−0.18	−0.32	−0.20	−0.30	−0.48	−0.40	**0.71**	−0.33	−0.28	−0.25	−0.35	−0.26	−0.35	−0.56	−0.53	‐
28. IT 76	0.11	0.04	0.18	−0.19	−0.02	0.17	0.17	0.17	−0.27	0.03	0.13	0.14	0.27	0.13	0.23	0.34	0.34	−0.31	**0.59**	0.18	0.18	0.26	0.18	0.25	0.37	0.41	−0.36

H, hippocampus; Vol, volume; H Vol_ICV, percentage hippocampal volume as a proportion of ICV; FA, fractional anisotropy; MD, mean diffusivity; LM, logical memory; VPA, verbal paired associates; SpS, Spatial Span; DSB, Digit Span Backward; LNS, Letter‐Number Sequencing; DSS, Digit‐Symbol Substitution; SSe, Symbol Search; CRT, Choice Reaction Time; IT, Inspection Time. Cells in bold type indicate the correlation of each measure at age 73 years with the same measure at age 76 years (the cross‐wave stability of hippocampal measurements and cognitive ability measurements).

### 3.1 Longitudinal change in hippocampal and cognitive measures

Mean hippocampal volume decreased by 132 mm^3^ between the two waves (a decrease of 0.90 standard deviations across the 3 years, *z *= −18.88, *p *<* *.001). Mean hippocampal volume as a proportion of ICV decreased by 0.03% between the two waves (0.99 SDs, *z* = −18.73, *p* < .001). Over the same period, hippocampal FA declined by 0.56 SDs (*z *= −13.66, *p *<* *.001), and hippocampal MD increased by 1.02 SDs (*z *=* *19.64, *p *<* *.001). Hippocampal T1 decreased significantly across waves, by 1.47 SDs (*z *= −18.90, *p *<* *.001). There were significant between‐wave mean changes in each of the three cognitive domains: verbal memory (decrease of 0.12 SDs; *z *= −3.48; *p *=* *.001), working memory (decrease of 0.21 SDs; *z *= −6.12, *p *<* *.001), and processing speed (decrease of 0.40 SDs; *z *= −12.48, *p *<* *.001).

### 3.2 Latent change score models of hippocampal volume and cognitive functions

We first tested the latent change score models' fit to the data using multiple absolute fit indices and the criteria suggested by Hu & Bentler (Hu & Bentler, 1999). The values are shown in Table 3. For all five hippocampal measures, the models showed excellent fit to the data.

**Table 3:** Absolute fit statistics for each of the latent change score models

Model	χ^2^	*df*	*p*	RMSEA	CFI	TLI
H Vol	644.84	279	<.001	0.039	0.964	0.958
H Vol_ICV	656.47	279	<.001	0.040	0.963	0.957
FA	682.16	279	<.001	0.041	0.961	0.954
MD	709.62	279	<.001	0.042	0.958	0.951
T1	710.03	279	<.001	0.042	0.957	0.950

RMSEA, root mean square error of approximation; CFI, comparative fit index; TLI, Tucker‐Lewis Index; H Vol, hippocampal volume; H Vol_ICV, percentage hippocampal volume corrected for ICV; FA, hippocampal fractional anisotropy; MD, hippocampal mean diffusivity; T1, hippocampal T1.

The key results from the latent change score models are shown in Table 4. First, the models indicated that, at baseline (“level‐level” correlations), the hippocampal variables were generally correlated with the cognitive domains in the expected direction. That is, higher volume was correlated with better cognitive abilities; however, for volume, the relation with processing speed was not significant, and none of the correlations between volume and cognitive ability were significant after multiple comparison correction. Higher FA, lower MD, and lower T1 were nominally significantly correlated with better cognitive abilities in all three domains; only the correlation between hippocampal FA and verbal memory did not survive FDR correction. Effect sizes were generally small (all absolute standardized estimates < 0.26). Many of these results, at the initial scanning/testing wave, have previously been reported by Aribisala et al. (Aribisala et al., 2014).

**Table 4:** Results from each of the latent change score models. Values are standardized path coefficients with SE in parentheses

Correlation type	Cognitive variable	H Vol	H Vol_ICV	FA	MD	T1
Level‐level (i.e., baseline measurements)	Working memory	0.**123 (0.046)** *, a	0.048 (0.047)	**0.184 (0.045)** ***	**−0.159 (0.045)** ***	**−0.156 (0.046)** **
	Verbal memory	**0.087 (0.040)** * a	0.045 (0.041)	**0.085 (0.040)** * a	**−0.170 (0.039)** ***	**−0.111 (0.040)** **
	Speed	0.070 (0.044)	**−**0.028 (0.045)	**0.234 (0.043)** ***	**−0.259 (0.042)** ***	**−0.174 (0.044)** ***
Cog. level‐hipp. change (i.e., baseline cognition predicting hippocampal change)	Working memory	0.040 (0.054)	0.040 (0.054)	**−**0.019 (0.054)	**−**0.011 (0.054)	0.030 (0.052)
	Verbal memory	0.088 (0.048)	**0.104 (0.048)** * a	**−**0.019 (0.049)	**−**0.040 (0.049)	0.019 (0.047)
	Speed	0.097 (0.053)	0.090 (0.053)	**−**0.016 (0.054)	**−**0.036 (.054)	0.017 (0.052)
Hipp. level‐cog. change (i.e., baseline hippocampal measures predicting cognitive change)	Working memory	**−**0.077 (0.080)	‐0.061 (0.081)	0.019 (0.080)	**−0.281 (0.080)** ***	0.039 (0.080)
	Verbal memory	0.078 (0.047)	**0.101 (0.034)** * a	0.085 (0.047)	**−0.150 (0.047)** ***	0.053 (0.079)
	Speed	0.096 (0.065)	**0.149 (0.065)** * a	0.044 (0.065)	**−0.197 (0.064)** **	**−**0.036 (0.064)
Change‐change (i.e., coupled changes)	Working memory	0.087 (0.086)	0.094 (0.087)	0.100 (0.088)	**0.194 (0.087)** *	**−0.167 (0.084)** * a
	Verbal memory	**−**0.008 (0.052)	**−**0.094 (0.087)	**−**0.061 (0.052)	**−**0.011 (0.052)	**−**0.091 (0.050)
	Speed	0.060 (0.071)	0.079 (0.071)	0.012 (0.072)	0.003 (0.072)	**−**0.056 (0.070)

Statistically significant values are in bold. *p <* *.05, **p <* *.01, ***p <* *.001. adid not survive FDR correction for multiple comparisons; all other statistically significant values remained so after correction. H Vol, hippocampal volume; H Vol_ICV, percentage hippocampal volume as a proportion of ICV; FA, hippocampal fractional anisotropy; MD, hippocampal mean diffusivity; T1, hippocampal T1.

We next examined “level‐change” correlations, first testing whether cognitive abilities at baseline predicted subsequent change in the hippocampal MRI biomarkers. None of these correlations were statistically significant for hippocampal volume, FA, MD, and T1. However, we did observe significant correlation for H_ICV: individuals with higher verbal memory at baseline exhibited less decline in H_ICV at follow‐up. However, this correlation was no longer significant after FDR correction.

We then tested the converse correlations: whether initial hippocampal MRI biomarker levels predicted subsequent change in cognitive abilities. None of these level‐change correlations were significant for hippocampal volume, FA, or T1. However, we did observe significant correlations for H_ICV and MD. Individuals with higher H_ICV at baseline experienced less decline in verbal memory and processing speed across the follow‐up. However, these correlations did not survive correction for multiple comparisons. Individuals with higher (putatively less healthy) hippocampal MD at baseline had more subsequent decline in all three of the cognitive domains measured here (standardized estimates = 0.28, 0.15, and 0.20 for working memory, verbal memory, and speed, respectively), and these relationships survived FDR correction.

Finally, we examined whether there was coupled change in the hippocampal and cognitive variables (i.e., “change‐change” correlations). All of these were nonsignificant, except for two, between MD change and change in working memory, and between T1 change and change in working memory. The former correlation was not in the expected direction; the result showed that greater increases in MD were related to less decline in working memory. This unexpected correlation was small in effect size (standardized estimate = 0.19, *p *=* *.03), but survived multiple comparisons correction. The latter change‐change correlation, between T1 and working memory, was significant in the uncorrected model (standardized estimate = −0.09, *p *=* *.047), but did not survive correction for multiple comparisons.

Finally, because some of the participants in the study may have been suffering from significant cognitive impairment (e.g., dementia), we excluded all of those individuals who had scored below 24, a commonly used cutoff point indicating possible pathological cognitive aging, on the MMSE (Folstein et al., 1975). Excluding the 19 individuals who scored below the cutoff at one or more of the three waves of the study made little difference to the results; there were only small differences in the regression parameters reported in Table 4 and the substantive conclusions remained the same.

## 4 DISCUSSION

To the best of our knowledge, this is the first longitudinal study to investigate associations between multiple measures of hippocampal integrity and cognitive functions in a large sample of older adults. The principal new findings that survived correction for multiple testing were that individuals with higher hippocampal MD (considered less healthy) at age 73 years, displayed subsequent decline in working memory, verbal memory, and processing speed. Other hippocampal parameters were not significant predictors of cognitive decline.

In a previous analyses of a cross‐sectional sample from the LBC1936 at age 73 that included 565 participants (Aribisala et al., 2014), we investigated whether there were associations between magnetization transfer ratio (MTR), FA, MD, and T1 with general factors of fluid type intelligence (*g*), cognitive processing speed, and memory. In this study, we have expanded the number of participants used in the investigation at 73 years (*N* = 655) and investigated the longitudinal relationship between hippocampal MRI biomarkers in verbal memory, working memory, and information processing speed. MTR measures are not presented in this study because it shows anomalous results at third wave that require further investigation. We subcategorized memory to verbal memory and working memory based on hippocampus' role in memory. We also used latent variable modeling instead of multivariate regression models to minimize cognitive test‐specific measurement error.

Our findings of baseline associations between hippocampal volume and memory are consistent with previous studies (Erickson et al., 2010; van der Lijn et al., 2008; Ystad et al., 2009), although in this sample, these did not survive FDR correction. Our study also found that higher FA and lower MD values in the hippocampus were associated with better cognitive abilities, and this is consistent with previous studies (Carlesimo, Cherubini, Caltagirone, & Spalletta, 2010; den Heijer et al., 2012; Müller et al., 2005). Again, the association between FA and verbal memory did not survive multiple testing correction. These associations are similar to our previous cross‐sectional study (Aribisala et al., 2014), where higher MD was significantly associated with lower scores of *g*, speed, and memory, while higher hippocampal FA were significantly associated with higher scores of *g* and speed, but not memory. We also observed a significant association between poorer performance in cognitive variables and higher T1 at baseline. Again, this finding is in agreement with our previous work (Aribisala et al., 2014), where higher T1 was significantly associated with lower scores of *g*, speed, and memory. This finding concerning MD and T1 suggests that hippocampal structure may undergo an age‐related increase in tissue water content (Cho et al., 1997; Gideon, Thomsen, & Henriksen, 1994). All of these observations detected using quantitative MRI techniques are reflective of microstructural changes at the cellular level during aging that may have begun to affect cognitive functioning, before changes in volume are detected.

None of the cognitive measures at age 73 years predicted changes in hippocampal MRI biomarkers between ages 73 and 76, and neither hippocampal volume, H_ICV, FA, and T1 predicted the cognitive change in this period. However, baseline hippocampal MD predicted 3‐year changes in verbal memory, working memory, and processing speed. Cross‐sectional studies (Carlesimo et al., 2010; den Heijer et al., 2012) have reported higher hippocampal MD being associated with poorer cognition. However, to the best of our knowledge, and for the first time, a study on a large aging sample of cognitively normal individuals shows that increasing water molecules' mobility predicts a steeper decline in all these three cognitive domains. It is also broadly consistent with the finding that skeletonized whole‐brain white matter MD has the greatest sensitivity for concurrent cognitive ability in patients with small vessel and Alzheimer's disease (Baykara et al., 2016).

Our analysis of correlations in coupled changes identified that increasing MD between 73 and 76 is associated with less decline in working memory. This finding was unexpected, given that increased MD is thought to partly reflect older age‐related changes in water content, disruption, and break down of tissue cytoarchitecture and demyelination that is associated with poorer memory (Beaulieu, 2002; Bhagat & Beaulieu, 2004; den Heijer et al., 2012; Hsu et al., 2010; McDonald et al., 2008; Neil et al., 2002; Pal et al., 2011). However, MD is also influenced by several other microstructural properties in the brain, and variations in these are highly dynamic. Further investigation is needed to compare differences in patients and healthy participants in clinical studies to understand the variability in hippocampal MD and subtle fluctuations in working memory and to exclude a survivor bias. No other coupled changes between any of the hippocampal and cognitive variables survived multiple testing correction.

The significant decrease in hippocampal T1 between 73 and 76 was somewhat unexpected and should be interpreted with caution. Our finding was not linked to any potential differences in data acquisition or preprocessing between waves 2 and 3. We include Figure S1 to demonstrate that there was a decline in T1 during the 3‐year period of the data collection of wave 3, suggesting a real decline of T1 at the seventh decade of life. T1 signal has previously been shown to be influenced by scanner drift (Armitage, Farrall, Carpenter, Doubal, & Wardlaw, 2011), but there was little evidence of significant drift in our regular quality assurance data. A previous study showed that areas of deep gray matter are prone to iron accumulation with aging (Lim et al., 2013) which shortens T1 (Ogg & Steen, 1998). A similar process could account for the T1 decrease in this cohort, although we have not tested for iron accumulation in the hippocampal region.

In addition to the associations found between hippocampal volume and cognitive measures, the variation in the associations between quantitative hippocampal MRI measures and cognitive performance may indicate that quantitative MRI biomarkers are sensitive at detecting histopathological changes, allowing us to study the cellular changes underpinning age‐related tissue loss at the seventh decade of life. Previous studies have shown that MD, FA, and T1 (Bastin et al., 2002; Bhagat & Beaulieu, 2004; Cho et al., 1997; den Heijer et al., 2012; Hong et al., 2010; Hsu et al., 2010; Neil et al., 2002; Pal et al., 2011) differ between various patient groups, age, and gender, making these biomarkers ideal for distinguishing subtle differences in the underlying pathology of diseases with overlapping characteristics, such as dementia, Alzheimer's, multiple sclerosis, and Parkinson's disease. This strengthens the use of multimodal MRI in studying age‐related structural changes in large longitudinal or cross‐sectional dataset of normal aging population. Information on cognitive abilities included in the analysis of the multimodal MRI measures, will hopefully lead to clearer understanding of the underlying mechanisms influencing cognitive outcomes.

The main limitation of this study is that our results may not be fully generalizable since our population sample is self‐selected, increasing the likelihood of participants who are healthier and have a higher cognitive ability and probably less variance compared to similarly aged individuals in the general population. Therefore, they may be showing comparatively modest hippocampal and cognitive decline, relative to the population. Thus, the associations here are likely to be conservative estimates of coupled hippocampal integrity and cognitive functions changes. In addition, participants who did not return for a second MRI scan had significantly lower cognitive ability measures compared to participants who returned. This suggests that there were restrictions in the range of participants in the latter wave and, therefore, the correlations may be somewhat stronger in a fully representative sample. In a previous dropout analysis in this dataset, participants with higher baseline levels of cognitive ability were shown to be more likely to return at the third wave, and a large variety of medical, social, and physical measurements taken at baseline did not improve significantly upon this prediction of study attrition (Ritchie et al., 2016). Thus, either data were missing at random or were missing due to variables that were not included in this dropout analysis.

This study is broadly focused on healthy participants and, therefore, it does not directly address participants with Alzheimer's disease, other dementias, and aging‐related neuropathologies. Nonetheless, it provides us with important information for understanding nonpathological‐based aging‐related cognitive decline (Boyle et al., 2013), that could potentially assist in diagnosing early stages of any aging‐related neuropathologies, because in some cases, accelerated decline could predict pathologies (Mura et al., 2014). The 3‐year follow‐up period may be too short to find significant associations between the changes in hippocampal integrity and cognitive functions in the seventh decade of life. Since simulations have shown that the power to detect correlated changes between variables in longitudinal studies increases substantially with greater follow‐up durations (Rast & Hofer, 2014), these associations could be improved when data from a further 3‐year follow‐up (thus 6 years from initial scanning) become available; the fourth wave is underway. Finally, it should be noted that the hippocampal measures presented here could in fact be reflecting the whole‐brain correlations and may not be truly hippocampus specific.

The strength of this work is that our data come from a longitudinal study containing detailed neuroimaging measures of the hippocampus alongside a wide range of cognitive tests undertaken by the participants. These allowed us to investigate the associations between multiple measures of hippocampal integrity and cognitive functions, rather than using gross hippocampal volumetric measurements alone. Using a large sample with narrow age range, we minimized potential risk confounding in between‐person and within‐person age differences (Hofer & Sliwinski, 2001). Our use of Latent Difference Score model (McArdle, 2009) also allowed error‐free estimates of longitudinal changes in the hippocampal structure and cognitive abilities. Future studies of the association between hippocampal integrity and cognitive aging should take into account other additional indicators of brain health, such as vascular disease, global atrophy and loss of tissue in specific brain structures, neuronal morphology, mineralization dysregulation, and gene expression variation, since all of these candidates can feasibly explain variations in the aging of cognitive functions.

The present analysis of coupled changes correlations add to our earlier finding of high MD at age 73 being sensitive to concurrent cognitive function and suggest that increasing MD between 73 and 76 is associated with less decline in working memory; the latter requires further investigation. We found no other coupled changes between any of the hippocampal and cognitive measures. Advanced quantitative MRI techniques such as diffusion tensor MRI and relaxometry may therefore be more useful in determining age‐related microstructural changes in the hippocampus than volume.

## CONFLICT OF INTEREST

None.
