# Supplementary figures and images for: Coupled changes in hippocampal structure and cognitive ability in later life
Source: Brain Behav. 2018 Jan 4;8(2):e00838. doi: 10.1002/brb3.838 (PMC5822578; doi:10.1002/brb3.838)

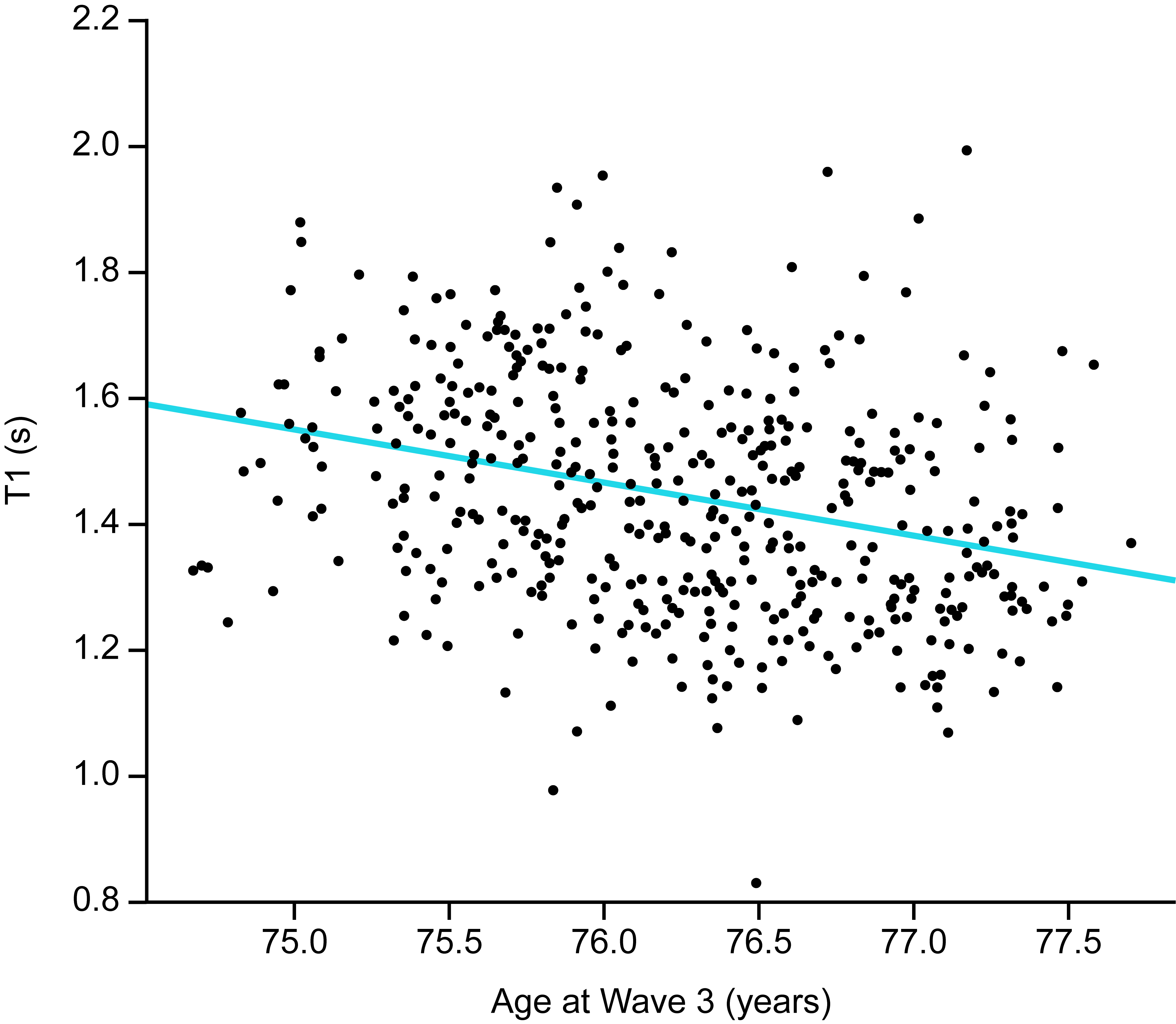

Supplement: Supplementary file 1 [file BRB3-8-e00838-s001.tif]
